# Gene expression profiling identifies FLT3 mutation-like cases in wild-type FLT3 acute myeloid leukemia

**DOI:** 10.1371/journal.pone.0247093

**Published:** 2021-02-16

**Authors:** Adrián Mosquera Orgueira, Andrés Peleteiro Raíndo, Miguel Cid López, Beatriz Antelo Rodríguez, José Ángel Díaz Arias, Roi Ferreiro Ferro, Natalia Alonso Vence, Ángeles Bendaña López, Aitor Abuín Blanco, Laura Bao Pérez, Paula Melero Valentín, Marta Sonia González Pérez, Claudio Cerchione, Giovanni Martinelli, Pau Montesinos Fernández, Manuel Mateo Pérez Encinas, José Luis Bello López

**Affiliations:** 1 Health Research Institute of Santiago de Compostela (IDIS), Santiago, Spain; 2 Division of Hematology, Complexo Hospitalario Universitario de Santiago de Compostela (CHUS), SERGAS, Santiago, Spain; 3 University of Santiago de Compostela, Santiago, Spain; 4 University of Bologna, Bologna, Italy; 5 Istituto Scientifico Romagnolo per lo Studio e la Cura dei Tumori, Meldola, Italy; 6 University Hospital of La Fe in Valencia, Valencia, Spain; European Institute of Oncology, ITALY

## Abstract

**Background:**

*FLT3* mutation is present in 25–30% of all acute myeloid leukemias (AML), and it is associated with adverse outcome. FLT3 inhibitors have shown improved survival results in AML both as upfront treatment and in relapsed/refractory disease. Curiously, a variable proportion of wild-type *FLT3* patients also responded to these drugs.

**Methods:**

We analyzed 6 different transcriptomic datasets of AML cases. Differential expression between mutated and wild-type *FLT3* AMLs was performed with the Wilcoxon-rank sum test. Hierarchical clustering was used to identify *FLT3*-mutation like AMLs. Finally, enrichment in recurrent mutations was performed with the Fisher’s test.

**Results:**

A *FLT3* mutation-like gene expression pattern was identified among wild-type *FLT3* AMLs. This pattern was highly enriched in *NPM1* and *DNMT3A* mutants, and particularly in combined *NPM1*/*DNMT3A* mutants.

**Conclusions:**

We identified a *FLT3* mutation-like gene expression pattern in AML which was highly enriched in *NPM1* and *DNMT3A* mutations. Future analysis about the predictive role of this biomarker among wild-type *FLT3* patients treated with FLT3 inhibitors is envisaged.

## 1. Introduction

FMS-like tyrosine kinase-3 (FLT3) is a receptor tyrosine kinase commonly mutated in acute myeloid leukemia (AML) [1]. *FLT3 internal tandem duplication* (ITD) is the most common mutation, affecting 25% of de novo AML cases [2], and additional tyrosine kinase mutations (*FLT3*-TKD) in codons D385 and I386 have been observed in 5–10% of cases [3]. Both types of mutations induce constitutive activation of FLT3 kinase activity, inducing pro-survival and proliferative signals [4]. *FLT3*-ITD mutation confers a poor outcome in de novo and relapsed AML, and it is currently suggested to include these patients in clinical trials [5, 6].

A number of promising FLT3 inhibitors are under development for AML treatment. The addition of the multi-kinase inhibitor midostaurin [7] to standard chemotherapy in newly diagnosed *FLT3*-mutated (*FLT3*^*mut*^) AML significantly improved event-free survival and overall survival in all *FLT3* mutation subtypes [8]. Several novel FLT3 inhibitors with increased specificity for FLT3 are under study, such as gilteritinib, crenolanib and quizartinib [3], with encouraging results from phase 2 and 3 trials in the relapsed & refractory AML setting [9–11]. Curiously, overall responses among wild-type *FLT3* AML patients were reported for both gilteritinib (12% of composite overall responses) and quizartinib (30–36% of composite complete remissions) [9, 11]. It has been hypothesized that this effect could be due to nonspecific inhibition of other kinases, cryptic FLT3 activation by other mutations or hyperactivation of FLT3 by its ligand [9]. Not surprisingly, 2 clinical trials are currently underway in order to study the activity of FLT3 inhibitors in wild-type FLT3 patients [12, 13], but there is still no biomarker to predict which patients will respond to these drugs. Therefore, an interesting point would be to identify gene expression changes associated with *FLT3* mutation in order to search for wild-type *FLT3* cases that resemble *FLT3*^*mut*^ AMLs at the transcriptomic level.

In this study we analyzed the transcriptomic pattern of 6 different AML cohorts, which enabled the identification of a *FLT3* mutation-like gene expression pattern highly enriched in *NPM1* and *DNMT3A* mutants. Our results indicate common deregulated pathways among these leukemias, opening the way to study the role of this biomarker in order to predict responses to FLT3 inhibitors in wild-type *FLT3* AML.

## 2. Materials and methods

We analyzed five AML gene expression datasets available in the *Gene Expression Omnibus* (GEO): GSE14468 (461 cases of de novo cytogenetically normal AML), GSE10358 (188 cases of de novo AML), GSE61804 (279 de novo AML cases), GSE17855 (237 pediatric AML cases) and GSE15434 (251 cases of normal karyotype AML). In the case of GSE14468 and GSE10358, both *FLT3*-ITD and D385 mutation status were reported, whereas in the remaining datasets only the *FLT3*-ITD status was informed. All datasets used the same type of gene expression arrays: *Affymetrix Human Genome U133 Plus 2*.*0 Array*. Signal intensities were rank-normalized, and differential expression between *FLT3*^*mut*^ (either ITD or TKD) and wild-type samples was performed in the largest cohort (GSE14468) using the two-sided Wilcoxon-rank sum test. P-values were adjusted for multiple testing using the Bonferroni method. Hierarchical clusterization and heatmap plots were created using the *heatmaps3* package with default parameters [14], and enrichment analysis was performed with the two-sided exact Fisher’s test. Gene ontology analysis was performed on *WebGestalt* with default parameters (FDR significance threshold, 0.05) [15].

RNAseq and mutation data from 246 AML patients treated with intensive chemotherapy was retrieved from *Bamopoulos et al*. (GEO identification GSE146173) [16]. Raw count data was transformed to normalized transcripts per million using the *fpkm* function implemented in the *DESEQ2* package [17]. Afterwards, rank-transformation was applied. 595 genes matching genes with the *FLT3*-like pattern were selected, followed by standard hierarchical clusterization. Differential mutation between the *FLT3*-like and no-*FLT3*-like group was performed with Fisher’s test.

All computations except gene ontology analysis were performed in R. All data used for this analysis is readily accessible from public repositories.

## 3. Results

### *FLT3*-like gene expression pattern

**Fig 1:**
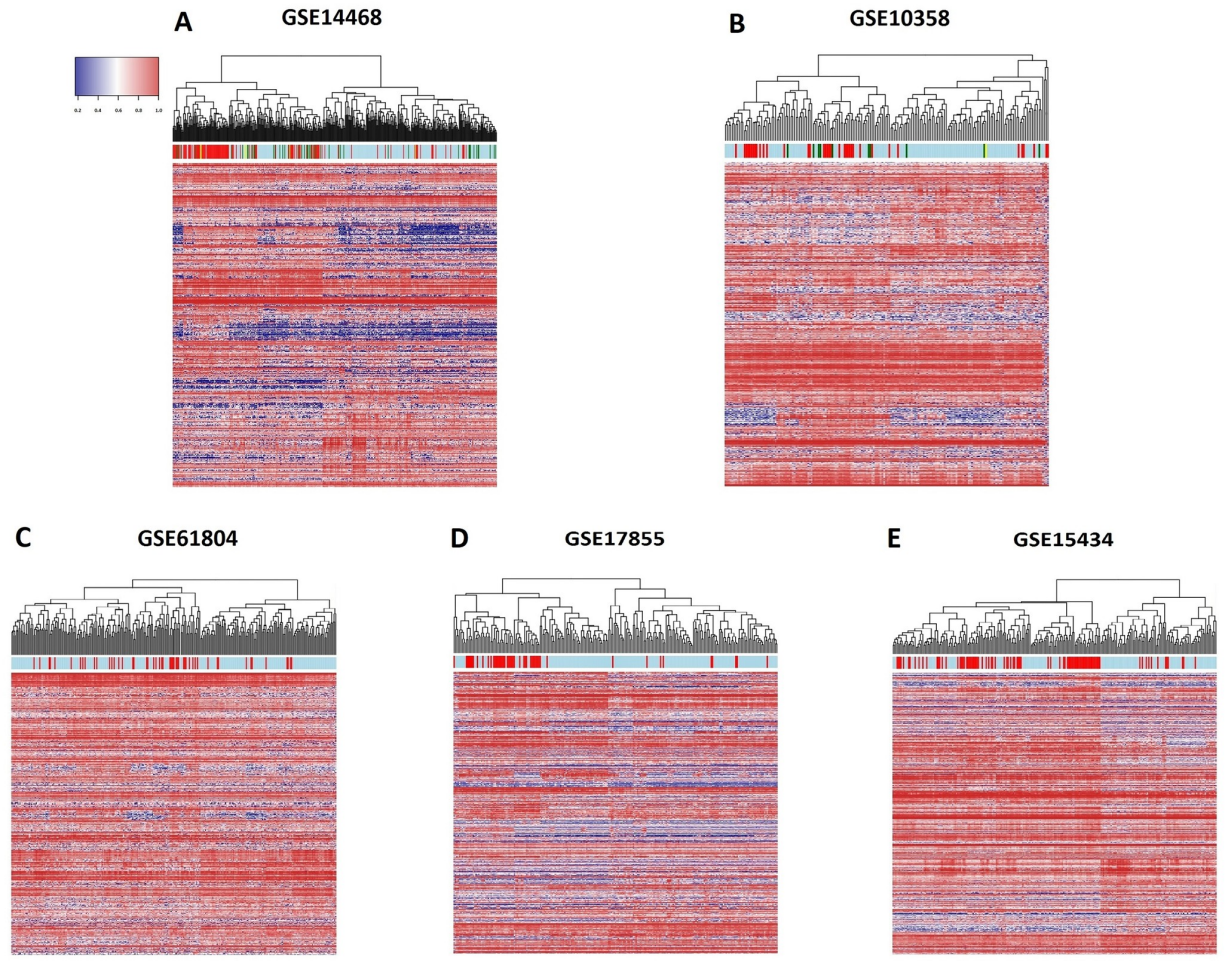
**A and B)** Heatmaps representing the hierarchical clustering of AML cases according to the expression of the 911 probes associated with *FLT3* ITD and TKD mutation status. The color coding of the bar indicates if a patient harbos a *FLT3*-ITD mutation (red bars), a *FLT3*-TKD mutation (green bars), both mutations (yellow bars) or no *FLT3* mutation (light blue bars). **C-E)** Heatmaps representing the hierarchical clustering of AML cases according to the expression of the 911 probes associated with *FLT3*-ITD mutation status. No information about *FLT3*-TKD mutations was reported for patients in these databases. Red bars indicate *FLT3*-ITD cases and light blue bars indicate lack of *FLT3*-ITD mutation.

We chose the largest database (GSE14468) as the discovery set, and we identified 911 probes mapping to 649 different genes which were differentially expressed between *FLT3*^*mut*^ (ITD and/or TKD) and wild-type *FLT3* samples (Bonferroni p-value <0.05, S1 Table). None of the probes mapped to *FLT3*. These probes were significantly enriched in 52 gene ontology terms, among which 17 terms were associated with hemopoiesis & immunology, and 3 terms were specifically linked to myeloid differentiation, namely *myeloid cell homeostasis*, q-value 5.01 x 10^−3^; *myeloid cell differentiation*, q-value 4.86 x 10^−3^; and *negative regulation of myeloid cell differentiation*, q-value 0.03 (S2 Table). Hierarchical clusterization revealed two broad groups. A cluster of 46.20% of patients was substantially enriched in *FLT3* mutants (p-value < 1 x 10^−4^), since it contained 81.67% of all *FLT3*-ITD cases, 61.36% of all *FLT3*-TKD cases and 83.33 of composed mutants (*FLT3*-ITD plus *FLT3*-TKD; Fig 1A). 28.52% of wild-type *FLT3* cases were also clustered within this group. A similar finding was identified in the independent GSE10358 database, where a cluster of 51.06% of patients contained 81.08% of all *FLT3*-ITD mutants and 70.00% of all *FLT3*-TKD mutants (p-value < 1 x 10^−4^). Furthermore, 42.15% of wild-type *FLT3* cases were also clustered within this group (Fig 1B).

**Fig 2:**
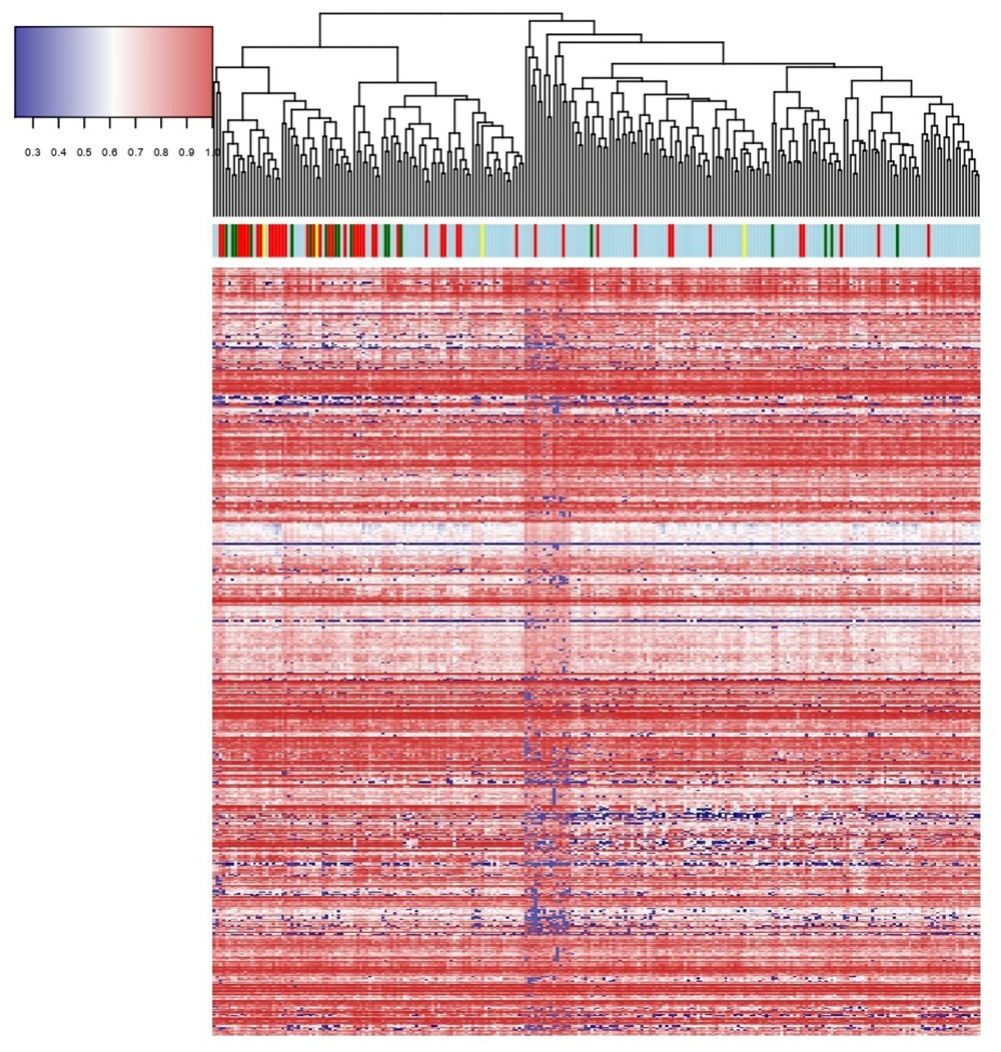
Heatmaps representing the hierarchical clustering of AML cases according to the expression of the 595 matching genes in the RNAseq cohort (GSE146173). The color coding of the bar follows the same pattern as that of Fig 1.

The same clustering was repeated in 3 different datasets that only reported *FLT3*-ITD mutation status. In GSE61804 a cluster comprising 58.06% of all patients harbored 78% of all *FLT3*-ITD cases (p-value 1.50 x 10^−3^), and 53.71% of all non *FLT3*-ITD cases were grouped within this cluster (Fig 1C). In GSE17855, a cluster of 47.67% of patients contained 81.25% of all *FLT3*-ITD patients (p-value < 1 x 10^−4^), but it also included 39.15% of all non *FLT3*-ITD cases (Fig 1D). Finally, in GSE15434, a cluster of 64.14% of patients was enriched in *FLT3*-ITD cases, comprising 86.67% of the whole cohort (p-value < 1 x 10^−4^); and additionally 49.11% of all non *FLT3*-ITD cases co-clustered within this group (Fig 1E). We replicated the *FLT3*-like pattern in GSE146173 using RNAseq data, identifying 28.49% of wild-type *FLT3* AMLs as *FLT3*-like (Fig 2).

### Mutation landscape of *FLT3*-like leukemias

Four of the microarray databases provided data about driver mutations in a few genes (S3 Table). The most significant finding was the enrichment of *FLT3*-like AMLs in *NPM1* mutants, which was observed in the 4 cohorts. This proportion was variable, ranging from 80.72% in karyotype normal AML (GSE15434) to just 12.16% in pediatric AML (GSE17855), indicating that the existence of the *FLT3*-like pattern is not explained by co-occurring *NPM1* mutations. Additionally, a relative enrichment in *IDH1* mutants within *FLT3*-like leukemias was detected in the discovery set GSE14468 (p-value 2.07 x 10^−4^). Furthermore, depletion of *CEBPA* mutants in *FLT3*-like AML was a common phenomenon in all 3 cohorts that reported mutations in this gene, and a reduced frequency of *KIT* mutations was also discovered in the pediatric AML dataset (GSE17855).

Using the most recent RNAseq data published by *Bamopoulos et al*. [16] we observed that *FLT3*-like leukemias were highly enriched in concurrent *NPM1* and *DNMT3A* mutants, whereas 19.60% of *FLT3*-like AMLs were *NPM1* and *DNMT3A* wild-type. Additionally, there was a tendency for an enrichment in *IDH1* mutations (p-value 0.09). On the contrary, *FLT3*-like AMLs were depleted in *ASXL1*, *CEBPA*, *RUNX1*, *TP53*, *SF3B1* and *U2AF1* mutations (Table 1).

**Table 1:** Differential distribution of recurrently mutated genes in wild-type *FLT3* AML patients according to the presence or absence of the *FLT3*-like pattern (GSE146173).

Gene ID	p-value	FLT3-like (%)	No FLT3-like (%)
*ASXL1*	5.29E-04	1.96	21.87
*CEBPA*	2.66E-15	0	5.47
*DNMT3A*	1.61E-03	41.17	17.19
*EZH2*	1	1.96	1.56
*IDH1*	0.09	15.69	7.03
*IDH2*	0.81	13.73	12.5
*NPM1*	3.40E-24	74.51	2.34
*RUNX1*	2.30E-05	1.96	28.12
*TET2*	0.29	23.53	16.41
*TP53*	0.02	1.96	14.06
*SRSF2*	0.62	9.8	13.28
*U2AF1*	7.95E-14	0	7.03
*SF3B1*	8.10E-18	0	3.12
*Concurrent NPM1 & DNMT3A*	7.11E-05	19.61	1.56

## 4. Discussion

In this report we describe the existence of a *FLT3* mutation-like gene expression pattern in wild-type *FLT3* AMLs. The *FLT3*-mutation like pattern was enriched in *NPM1* and *DNMT3A* mutant leukemias. This is probably related to the significant co-occurrence of *FLT3* mutations with those of *NPM1* and *DNMT3A* [18], which probably leads to partially overlapping transcriptomic fingerprints. Not surprisingly, the *FLT3*-like pattern contains numerous *HOX* genes, which have been previously vinculated with the *NPM1*-transcriptional pattern [19]. Nevertheless, the heterogeneous frequency of *NPM1* mutations among *FLT3*-like leukemias, and particularly its low frequency in pediatric AML, along with the reproducibility of the *FLT3*-like pattern in all cohorts, indicate that co-occurring *NPM1* mutations are insufficient to explain the existence of the *FLT3*-like pattern.

Our results suggest that a group of AMLs with *FLT3* plus *NPM1* and/or *DNMT3A* mutations share a similar transcriptomic background. Noteworthy, responses to the FLT3 inhibitor gilteritinib among relapsed *FLT3*^mut^ AMLs are superior in those patients with mutations in *NPM1* or *DNMT3A*, and particularly in those with both genes mutated [20], and similar findings were reported with crenolanib [21]. Furthermore, *FLT3-ITD* leukemias with mutations in *NPM1* or *DNMT3A* exhibit a different drug response mechanism to the FLT3 inhibitor quizartinib, where the cell differentiation effect predominates over the cytotoxic mechanism [22].

The use of FLT3 inhibitors in wild-type *FLT3* AML has been tested in some trials. For example, the FLT3 inhibitor midostaurin evidenced blast reduction responses in 53% of relapsed & refractory AML cases [23]. Similarly, responses to novel FLT3 inhibitors in a variable proportion of wild-type *FLT3* cases have been observed in phase I & II trials [24–26]. This has motivated the development of new studies to specifically test the possible benefit of adding these drugs in the upfront treatment for AML [27, 28]. As it is expected that only a subgroup of patients might benefit from FLT3 inhibitors, it is imperative to develop and test new predictive biomarkers of response. Therefore, it becomes necessary to test the predictive value of the *FLT3*-like pattern in these clinical trials. Additionally, the enrichment of *FLT3*-like leukemias in *IDH1* mutations (which are correlated with *NPM1* mutation [29]) could set the basis for the development of new clinical trials testing the combination of different check-point inhibitors in AML [30].

This study has some limitations. Firstly, a variable proportion of *FLT3* mutation-like samples were clustered near *FLT3* mutants across the different cohorts, which probably reflects substantial heterogeneity between them. One of the possible explanations for this could be a differential distribution of *NPM1* and *DNMT3A* mutants, since these are drivers of cytogenetically-normal AMLs (such as in the case of GSE15434) [31]. Finally, some of the datasets had only *FLT3*-ITD annotation, and a minority of the *FLT3*-like cases might indeed have *FLT3*-TKD mutations.

## 5. Conclusions

Our results are concordant with the existence of a *FLT3* mutation-like transcriptomic pattern with a different mutational background which clusters a proportion of wild-type *FLT3* AMLs with *FLT3*^*mut*^ samples. These leukemias were highly enriched in *NPM1*, and particularly in composed *NPM1*/*DNMT3A* mutants, but *NPM1* status alone was insufficient to explain the existence of the *FLT3*-like pattern. The analysis of wild-type *FLT3* AML patients treated with FLT3 inhibitors in clinical trials is envisaged in order to study its possible role as a drug response biomarker.

## Supporting information

S1 Table Annotation of all probes significantly associated with *FLT3* mutation status in AML. (XLSX)

S2 Table Significantly enriched gene ontology (biological process) terms from the list of genes associated with *FLT3* mutation status. (XLSX)

S3 Table Differential distribution of recurrently mutated genes in wild-type *FLT3* AML wild-type according to the presence or absence of the *FLT3*-like pattern. Data reported for mutations analyzed in GSE14468, GSE10358, GSE15434 and GSE17855.
(XLSX)
