# Correction: Gene expression profiling identifies FLT3 mutation-like cases in wild-type FLT3 acute myeloid leukemia

**DOI:** 10.1371/journal.pone.0274984

**Published:** 2022-09-15

**Authors:** Adrián Mosquera Orgueira, Andrés Peleteiro Raíndo, Miguel Cid López, Beatriz Antelo Rodríguez, José Ángel Díaz Arias, Roi Ferreiro Ferro, Natalia Alonso Vence, Ángeles Bendaña López, Aitor Abuín Blanco, Laura Bao Pérez, Paula Melero Valentín, Marta Sonia González Pérez, Claudio Cerchione, Giovanni Martinelli, Pau Montesinos Fernández, Manuel Mateo Pérez Encinas, José Luis Bello López

There is an error in affiliation 4 for author Claudio Cerchione. The correct affiliation 4 is: Hematology Unit, IRCCS Istituto Romagnolo per lo Studio dei Tumori (IRST) "Dino Amadori", Meldola, Italy.

There is also an error in affiliation 5 for author Giovanni Martinelli. The correct affiliation 5 is: Scientific Directorate, IRCCS Istituto Romagnolo per lo Studio dei Tumori (IRST) "Dino Amadori", Meldola, Italy.
